# Case Report: A case of diabetes mellitus and pneumonitis induced by envafolimab treatment in small cell lung cancer

**DOI:** 10.3389/fimmu.2026.1708071

**Published:** 2026-03-20

**Authors:** Yanyan Li, Hongke Wang, Guochen Xing, Xiaodan Guo, Shanyong Yi

**Affiliations:** 1 Department of Oncology, Zhengzhou Central Hospital Affiliated to Zhengzhou University, Zhengzhou, China; 2 The 5th Department of Neurology, Zhengzhou Central Hospital Affiliated to Zhengzhou University, Zhengzhou, China

**Keywords:** diabetic ketosis, envafolimab, immune checkpoint inhibitor-induced diabetes mellitus, immune checkpoint inhibitor-induced pneumonitis, small cell lung cancer

## Abstract

In this paper, we report a case of sequential immune-related adverse events induced by envafolimab in a patient with small cell lung cancer (SCLC), including immune checkpoint inhibitor-associated diabetes mellitus (ICI-DM), diabetic ketosis (DK), and pneumonitis (CIP). A 63-year-old male with limited-stage SCLC received 4 cycles of etoposide plus carboplatin chemotherapy followed by radiotherapy, concurrent with maintenance immunotherapy using envafolimab. Eight months after initiating immunotherapy, the patient developed symptoms of dry mouth and excessive thirst. The clinical presentation was consistent with ICI-DM, though all diabetes-associated autoantibodies were negative. After achieving glycemic control with insulin, envafolimab was resumed. At 15 months, metastasis to the left submandibular region was identified and managed with localized iodine-125 seed implantation; envafolimab monotherapy was continued as maintenance treatment. Nineteen months after initiation of immunotherapy, the patient presented with chest tightness, shortness of breath, and dyspnea. Further diagnostic evaluation confirmed CIP and radiation pneumonitis, which improved following glucocorticoid therapy. Envafolimab was consequently suspended, and topotecan therapy was initiated for one month as second-line therapy, but was ultimately discontinued due to financial constraints. Six months after discontinuing topotecan, the patient was readmitted due to a progressively enlarging left submandibular metastases. Laboratory findings upon admission revealed DK, which had occurred due to self-discontinuation of insulin and a switch to oral hypoglycemic agents one week ago. During hospitalization, recurrent syncope of unknown origin occurred. After clinical improvement with supportive care, anti-tumor therapy with anlotinib was initiated as third-line therapy. Following envafolimab immunotherapy, the patient sequentially developed ICI-DM, DK and CIP. With prompt intervention, severe complications such as diabetic ketoacidosis (DKA), hyperosmolar hyperglycemic state, and respiratory failure were successfully avoided. This case underscores the importance of early recognition and management of immune-related adverse events (irAEs). The occurrence of DK after self-discontinuation of insulin highlights the necessity for long-term insulin therapy in ICI-DM. The patient remains alive with an overall survival exceeding 27 months, suggesting that ICI-DM may represent a potential prognostic biomarker for favorable outcomes in patients receiving immunotherapy.

## Introduction

Immunotherapy, particularly immune checkpoint inhibitors such as programmed death-1 (PD-1) and its ligand (PD-L1), has emerged as a revolutionary approach in cancer treatment, demonstrating significant efficacy across diverse malignancies (1). However, response rates remain relatively low, with only about 20% of patients deriving benefits (2). Compared to chemotherapy and radiotherapy, immunotherapy is associated with a significantly lower incidence of severe adverse events, which has contributed to its widespread clinical application. This has led to an intensified research for immune predictive biomarkers that can help identify which populations are more likely to respond positively to immunotherapy. With the expanding use of immunotherapy, rare but severe adverse events such as immune checkpoint inhibitor-associated diabetes mellitus (ICI-DM), myasthenia gravis, myocarditis, and acute interstitial nephritis are increasingly being reported (3–5). This imperative drives ongoing exploration of biomarkers to predict both treatment responders and susceptibility to immune-related adverse events (irAEs). Tumor exhibiting microsatellite instability-high (MSI-H), mismatch repair deficient (dMMR) and high PD-L1 expression tend to respond better to immunotherapy (6–8).

In November 2021, envafolimab received market approval in China from the National Medical Products Administration, making it the world’s first subcutaneous PD-L1 inhibitor (9). Envafolimab exhibits a favorable safety profile with generally manageable adverse effects (10). However, due to the relatively small sample size in pre-approval pivotal clinical trials, uncommon but severe adverse events necessitate vigilant monitoring. Here we present a case report of sequential ICI-DM, diabetic ketosis (DK) and immune checkpoint inhibitor-associated pneumonitis (CIP) induced by envafolimab in a patient with small cell lung cancer (SCLC).

## Case presentation

**Figure 1:**
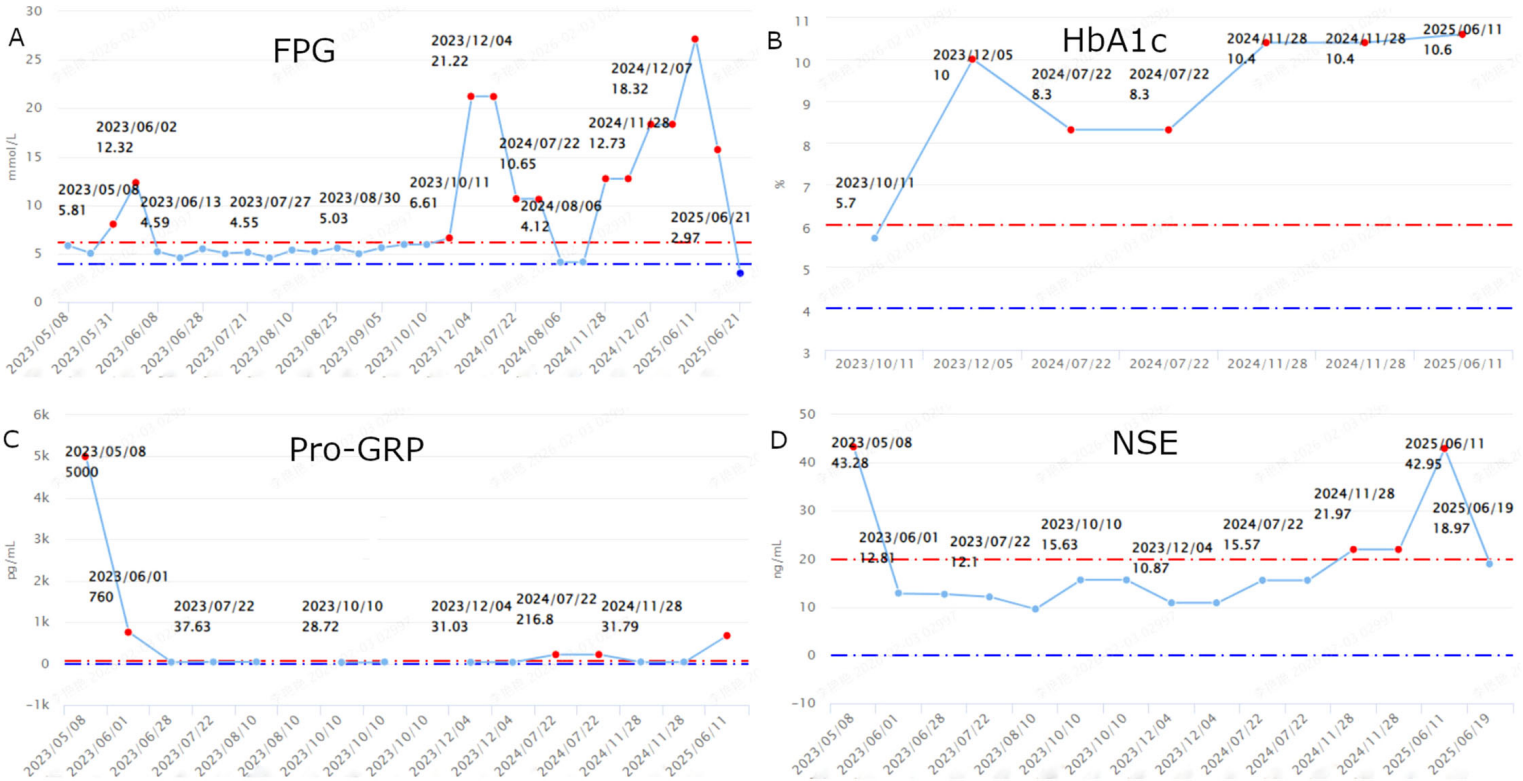
Line graphs showing longitudinal changes in **(A)** fasting plasma glucose (FPG), **(B)** glycated hemoglobin (HbA1c), **(C)** pro-gastrin-releasing peptide (Pro-GRP), and **(D)** neuron-specific enolase (NSE).

**Figure 2:**
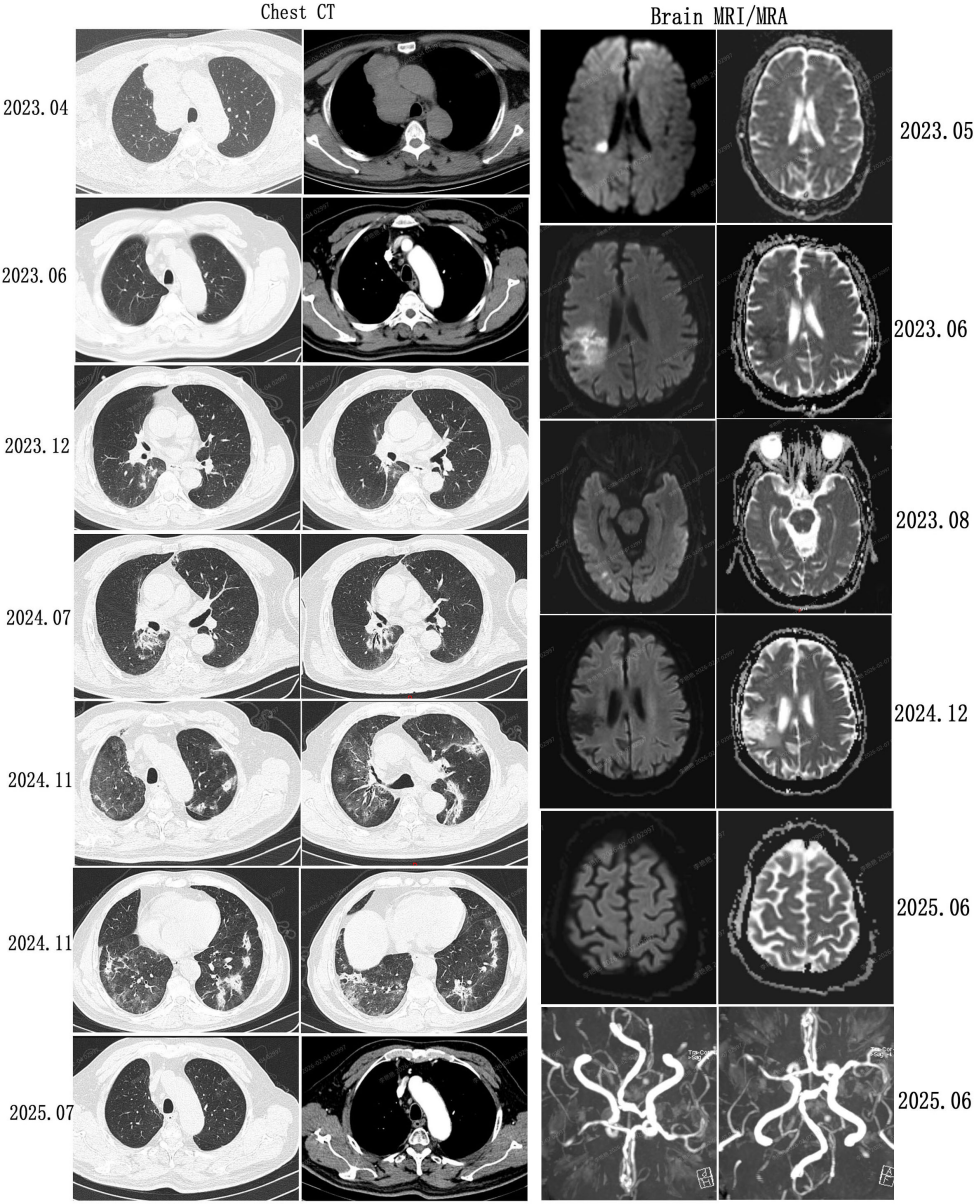
Representative serial images of chest CT, brain MRI, and brain MRA. Brain MRI sequences: diffusion-weighted imaging (DWI, left) shows a hyperintense lesion, and the corresponding apparent diffusion coefficient (ADC) map (right) demonstrates hypointensity, consistent with a subacute cerebral infarction. MRA reveals severe stenosis of the right middle cerebral artery.

In April 2023, a 63-year-old man (height: 170 cm; weight: 70 kg; BMI: 24.22 kg/m², above the normal range of 18.5 to 23.9 kg/m²) was admitted to our hospital for management of a newly diagnosed SCLC. Laboratory tests showed elevated levels of Pro-gastrin-releasing peptide (Pro-GRP; 5000 pg/mL, reference range: 0–70 pg/mL) (**Figure 1C**) and Neuron-Specific Enolase (NSE; 43.28 ng/mL, reference range: 0–20 ng/mL) (**Figure 1D**). He had a 40-year history of smoking with an average of 20 cigarettes per day and a 40-year history of alcohol consumption at 250g/day. He quit both smoking and alcohol immediately after the SCLC diagnosis. He had no history of occupational exposure. His medical history included hypertension for 8 years, which was well-controlled with medication, and a remote cerebral infarction 10 years previously without residual neurological deficits. He had no history of chronic obstructive pulmonary disease (COPD) or interstitial lung disease (ILD). He had no family history of diabetes mellitus. Notably, his father died of COPD with emphysema. Pathological slides from an outside hospital were reviewed at our hospital**’**s pathology department, confirming the diagnosis of SCLC. Contrast-enhanced brain Magnetic Resonance Imaging (MRI) and whole-body bone scan ruled out metastases to the brain and bones. Chest Computed Tomography (CT) revealed enlargement of the right pulmonary hilum and multiple mass shadows adjacent to the right hilum and in the mediastinum, with the largest cross-sectional dimension measuring 48 × 47 mm (**Figure 2**). The initial contrast-enhanced MRI of the liver revealed an abnormal signal in segment S8 with nodular enhancement in the arterial phase, in addition to hepatic cysts, raising suspicion of metastasis. The patient declined a liver biopsy for further clarification. His Eastern Cooperative Oncology Group (ECOG) performance status was 1. The planned treatment regimen was immunotherapy combined with platinum-based chemotherapy, followed by maintenance immunotherapy. The initial treatment consisted of envafolimab (150 mg SC weekly) in combination with etoposide (100 mg IV on D1–5 every 21 days) and carboplatin (100 mg IV on D1–5 every 21 days) for 4 cycles, followed maintenance monotherapy with weekly envafolimab. A sustained partial response(PR) was achieved after 2 cycles of treatment (**Figure 2**). Considering that the patient refused follow-up contrast-enhanced MRI after the 2nd and 4th treatment cycles, two subsequent contrast-enhanced CT scans of the upper abdomen showed only multiple hepatic cysts without abnormal enhancement in segment S8. Therefore, the likelihood of metastasis was deemed low, and the diagnosis of limited-stage SCLC (cT2N2M0 stage IIIB) was considered. We believed that sequential thoracic radiotherapy would likely provide clinical benefit to the patient. In August 2023, radiotherapy was initiated directed at the right hilar mass, metastatic mediastinal lymph nodes, and lymphatic drainage areas. The radiation schedule consisted of a clinical target volume dose of 45 Gy delivered in 30 fractions at 1.5 Gy per fraction, administered twice daily (10 fractions per week). To date, follow-up contrast-enhanced CT imaging has shown no definite abnormal enhancement in the liver.

On May 31, 2023, the patient was admitted due to chest pain prior to the second cycle of chemotherapy. Repeated tests showed normal B-type Natriuretic Peptide and troponin, while electrocardiogram and ambulatory electrocardiogram revealed ischemic ST-T changes. After consultation with the cardiology department, unstable angina was considered. The patient improved after treatment with isosorbide mononitrate tablets to dilate the coronary arteries and diltiazem to relieve coronary artery spasm. Markedly elevated random glucose and fasting plasma glucose (FPG) levels were attributed to stress-induced hyperglycemia, and no glucose-lowering intervention was administered. Follow-up testing six days later showed that FPG had returned to normal and remained stable thereafter (**Figure 1A**). In October 2023, the patient’s FPG measured 5.9 mmol/L(reference range: 3.9-6.1 mmol/L), while the glucose level measured using a glucometer on residual venous blood was 8.7 mmol/L. The following day, repeat testing showed a FPG of 6.61 mmol/L, and the glucose level measured again by glucometer on residual venous blood was 6.8 mmol/L, with a glycated hemoglobin (HbA1c) of 5.9% (reference range: 4-6%) (**Figure 1B**). Given the normal HbA1c level and the known potential for error in glucometer readings on residual venous blood, diabetes mellitus was not diagnosed. In December 2023, the patient presented with dry mouth and excessive thirst, which prompted readmission to the department of oncology. Admission laboratory tests revealed a FPG level of 21.22 mmol/L, continuous subcutaneous insulin infusion (CSII) was initiated following endocrinology consultation. Capillary blood glucose measurements on the same day ranged from 20.1 to 28.2 mmol/L. Additional laboratory investigations were performed. Arterial blood gas analysis and β-hydroxybutyrate levels were both within normal limits. HbA1c was 10% (**Figure 1B**). Fasting C-peptide was 0.92 ng/mL (reference range: 0.4-5.7 ng/mL), and postprandial C-peptide was 3.25 ng/mL. Serum insulin levels were not measured due to the patient’s ongoing use of CSII. Urinalysis showed no urinary ketones and glucose. Diabetes-associated autoantibodies—including glutamic acid decarboxylase 65 antibodies (GAD65), insulinoma-associated-2 antibodies (IA-2A), insulin autoantibodies (IAA), and islet cell cytoplasmic autoantibodies (ICA)—were all negative. Contrast-enhanced CT of the upper abdomen showed no abnormalities in the pancreas. The patient reported no abdominal pain, so amylase and lipase levels were not tested initially. Follow-up tests after six months showed that GAD65, IA-2A, and ICA antibodies remained negative, and lipase and amylase levels were within normal ranges. During hospitalization, glycemic control was maintained with CSII. Upon discharge, a basal-bolus regimen was prescribed: Insulin aspart (8-8–8 U) before each meal and Insulin glargine (22 U) at bedtime.

In July 2024, the patient was admitted with a one-month history of a progressive enlargement of the left submandibular mass. Laboratory tests revealed the following: FPG 10.65 mmol/L, fasting C-peptide 0.89 ng/mL, and HbA1c 8.3%. Fasting C-peptide levels showed a mild decline compared to the level of 7 months ago (0.92 ng/mL), indicating a reduction in basal insulin secretion capacity. Subsequently, an ultrasound-guided core needle biopsy of the left submandibular mass was performed. Integrated pathological and immunohistochemical analysis confirmed metastatic small cell carcinoma, while other lesions remained in partial response(PR). Local therapy was administered via implantation of 16 iodine-125(^125^I) radioactive seeds. Concurrently, immunotherapy with envafolimab was continued.

In December 2023, a chest CT revealed a newly emerged opacity in the right lower lobe (**Figure 2**). By July 2024, a follow-up chest CT showed persistent of this opacity, raising suspicion of radiation pneumonitis (RP) (**Figure 2**). As there were no signs of infection or respiratory symptoms, no specific intervention was undertaken. In November 2024, the patient presented with noticeable chest tightness and shortness of breath. A subsequent chest CT (**Figure 2**) indicated multiple scattered opacity in both lungs. Blood tests ruled out infectious diseases (**Table 1**). Given the diffuse bilateral distribution of these opacities extending beyond the radiation field, the findings were most consistent with a combination of CIP and RP. The patient was treated with methylprednisolone injection, which led to significant symptomatic improvement on the second day. The methylprednisolone dose was subsequently tapered gradually. After discharge, oral prednisone was administered and progressively reduced until discontinuation. Testing for ILD-associated autoantibodies was not performed. The main reasons include: 1) The patient exhibited no other symptoms suggestive of autoimmune diseases, such as rash, myalgia, weakness, arthralgia, fever, or Raynaud’s phenomenon. Moreover, no such symptoms emerged even more than six months after discontinuation of glucocorticoid therapy. 2) The patient began immunotherapy in May 2023 and radiotherapy in August 2023. The presence of multiple scattered abnormal opacities in both lungs, after excluding infection, was considered indicative of CIP combined with RP. 3) Respiratory symptoms improved rapidly following glucocorticoid therapy. Envafolimab was permanently discontinued. Second-line therapy with topotecan was initiated but discontinued after one month due to financial constraints.

**Table 1:** Labortatory findings.

Variables	2024.11	2025.06	Reference
Arterial Blood Gas Analysis			
pH	7.38	7.332	7.35-7.45
pO_2_, mmHg	63.5	101	80-100
pCO_2_ , mmHg	36.7	47.5	35-45
Bicarbonate, mmol/l	22	23.2	22-26
Anion Gap, mmol/l	10.7	8.1	8-16
SBE, mmol/l	-3.4	-0.8	-3~+3
Lactate, mmol/l	1.8	1	0.5-1.7
Venous blood parameters			
FBG, mmol/l	12.73	27.19	3.9-6.1
HbA1c, %	10.4	10.6	4.0-6.0
White Blood Cell, 10^9^/l	6.87	9.07	3.5-9.5
Neutrophil Percentage, %	71.8	92.1	40-75
Lymphocyte Percentage, %	14	3	20-50
C-Reactive Protein (CRP), mg/l	7.82	1.51	0-10
PCT, ng/ml	0.02	0.04	0-0.046
Interleukin-6 (IL-6), pg/ml	9	5.8	0-7
β-D-Glucan, pg/ml	<10	/	60-100
Galactomannan Antigen, ug/l	<0.1	/	<0.5
pro-BNP, pg/ml	/	154	300-900
Myoglobin, ng/ml	/	137	23-112
CK-MB, ng/ml	/	<2	2-7.2
Troponin, ng/ml	/	<0.01	0.01-0.023
β-hydroxybutyrate, mmol/l	/	0.65	0-0.3

On June 11, 2025, the patient was readmitted due to progressive enlargement of a left submandibular mass. Physical examination and imaging confirmed significant enlargement of the mass and regional lymph nodes, while other lesions remained in PR. Laboratory findings were as follows: FPG, 27.19 mmol/L; HbA1c, 10.6%; ProGRP, 675.37 pg/mL, β-hydroxybutyrate, 0.36 mmol/L (reference range: 0.03-0.3 mmol/L). The patient had self-discontinued insulin therapy 7 days prior to admission and switched to oral hypoglycemic agents. Insulin therapy was promptly resumed upon readmission. On June 15 at 06:00, the patient experienced a sudden loss of consciousness while using the toilet. Critical findings at that time include a blood glucose level of 10.1 mmol/L and a blood pressure of 187/117 mmHg. Emergency cranial CT showed no hemorrhage. Brain MRI revealed an acute cerebral infarction in the right parietal lobe, with severe stenosis or occlusion of the M2 segment and its distal branches of the right middle cerebral artery (MCA) (**Figure 2**). Following urgent neurology consultation, the patient was transferred to the neurology unit and received intravenous alteplase thrombolysis. Unfortunately, at 01:20 on June 19, the patient developed recurrent acute neurological deficits without identifiable triggers. Clinical manifestations included loss of consciousness, unresponsiveness to verbal stimuli, involuntary limb movements, and periodic apnea. A repeat head CT showed no hemorrhage. The patient was transferred to neurological intensive care unit (NICU). Sedation with midazolam was initiated but proved ineffective; therefore, sedation was escalated to ciprofol infusion. Relevant blood test results are presented in **Table 1**. Arterial blood gas analysis revealed a pH of 7.332. Although blood ketones were 0.65 mmol/L and blood glucose was 14.4 mmol/L, the partial pressure of carbon dioxide (PaCO_2_) was 45.7 mmHg, with bicarbonate, anion gap, and standard base excess all within normal ranges. This pattern was not consistent with diabetic ketoacidosis (DKA); instead, respiratory acidosis was considered the primary etiology. The basal-bolus insulin regimen was continued at the original dose. The patient’s neurological symptoms subsequently improved, and the acidosis resolved spontaneously, further supporting that DKA was not the underlying etiology.

**Figure 3:**
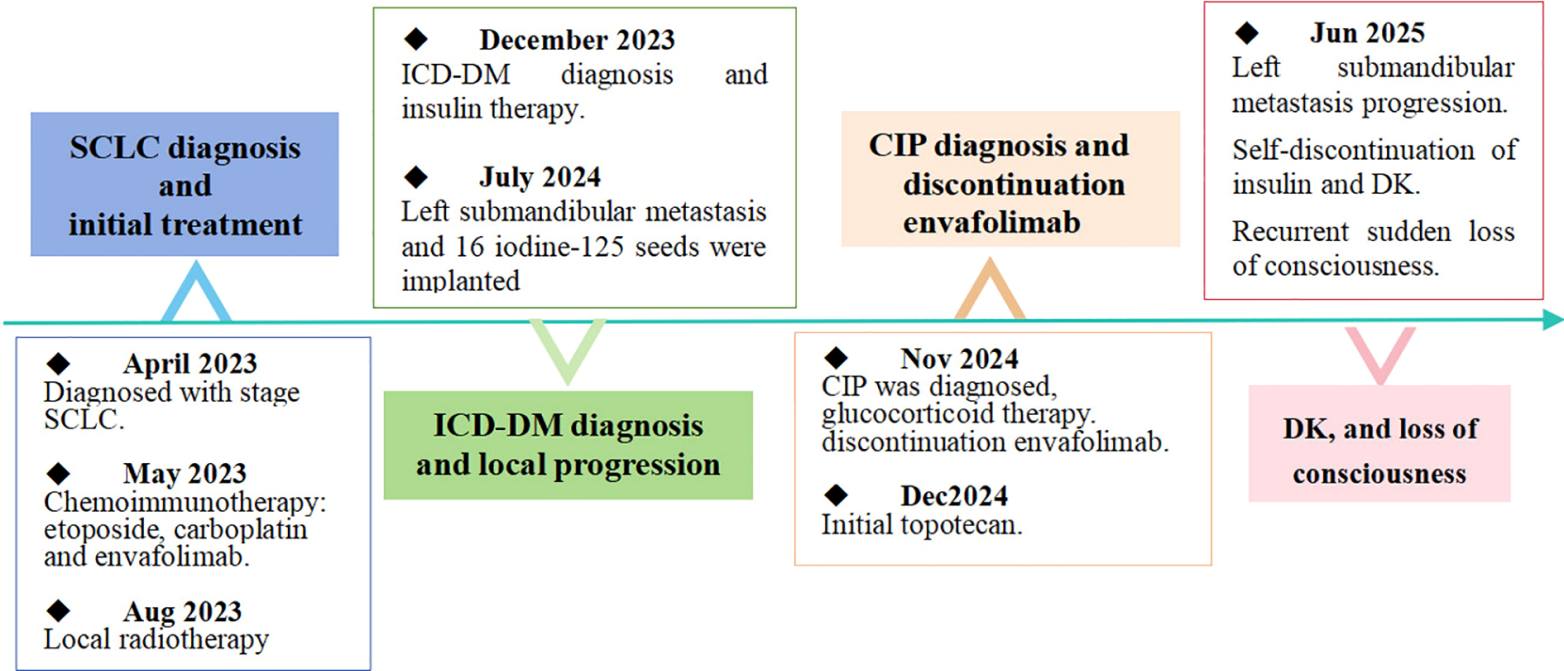
Timeline of disease progression, ICI-DM, CIP, RP, DK.

Brain MRI with Magnetic Resonance Angiography (MRA) and Susceptibility Weighted Imaging (SWI) revealed no hemorrhage or new infarction. Following initiation of antiepileptic therapy and cerebral perfusion, the patient gradually regained consciousness without residual motor deficits. Following an endocrine consultation on June 18 recommended a combination regimen of empagliflozin/metformin FDC, insulin aspart, and insulin glargine for glycemic control. Cerebrospinal fluid (CSF) routine and biochemical profiles were normal except an elevated total protein level of 878.9 mg/L(reference: 0–500 mg/L). All CSF smears (including routine bacterial smear, acid-fast bacillus smear for tuberculosis, and India ink smear for Cryptococcus neoformans) were normal. CSF cytology smear showed no malignant cells. CSF antibodies for Epstein-Barr virus, Coxsackievirus, Rubella virus, Cytomegalovirus, and Herpes simplex virus were all negative. The patient had not undergone baseline CSF screening prior to symptom onset. Since the SCLC diagnosis, multiple brain MRI scans have revealed no brain metastases. Despite the addition of statin to existing aspirin therapy, the patient subsequently experienced multiple episodes of asymptomatic subacute cerebral infarction (**Figure 2**). During hospitalizations, electrocardiograms showed no atrial fibrillation, and multiple echocardiograms ruled out valvular heart disease (a bubble study was not performed). Vascular assessments were first performed after the onset of neurological symptoms in June 2025 and revealed extensive atherosclerosis: uneven intima-media thickening with plaques in both carotid arteries (left bifurcation: 14.6 × 2.5 mm; right: 9.6 × 2.7 mm) and both subclavian arteries (left: 16.6 × 2 mm; right: 9.2 × 2.7 mm), along with transcranial Doppler findings suggestive of moderate-to-severe stenosis in the terminal internal carotid arteries and mild stenosis in the right anterior cerebral and basilar artery. The abdominal aorta and bilateral renal arteries were normal. The patient’s low-density lipoprotein cholesterol level had never been controlled below 1.8 mmol/L. Given to the recurrent episodes of loss of consciousness, a telemedicine consultation was requested from Beijing 301 Hospital. The external specialist suggested vasovagal syncope or alcohol withdrawal as possible etiologies, recommendations fall prevention strategies and supplementation with B vitamins (B12, B6, B1, and folic acid). For associated psychiatric symptoms, olanzapine orally disintegrating tablets and sodium valproate sustained-release tablets were prescribed. The progressive left submandibular mass indicated disease progression. After neurological stabilization, anlotinib was initiated as third-line therapy. The complete timeline from the time of diagnosis to June 2025 is shown in **Figure 3**.

## Discussion

Immune-related endocrine disorders are increasingly recognized, particularly ICI-DM. A comprehensive review of ICI-DM incidence reported the following rates: atezolizumab (an anti-PD-L1 antibody), 0.8% (52/6538); durvalumab (an anti-PD-L1 antibody), 0.26% (9/3447); nivolumab (an anti-PD-1 antibody), 0.47% (26/5520); pembrolizumab (an anti-PD-1 antibody), 0.51% (58/11403); ipilimumab (cytotoxic T-lymphocyte-associated antigen 4 inhibitor), 0.027% (1/3701); and dual immune checkpoint blockade therapy, nearly 1% (51/5116) (11). Although the incidence of ICI-DM is low, the rapid onset and potential to cause life-threatening DKA necessitate close monitoring (12). ICI-DM necessitates long-term insulin therapy. In our case, seven days after the patient self-discontinued insulin and switched to oral hypoglycemic agents, he developed DK.

In a phase II trial of envafolimab involving 103 patients with previously treated advanced dMMR/MSI-H solid tumors, endocrine disorders were the most common irAEs. Grade 3–4 toxicities occurred in 8% of patients, primarily manifesting as immune-related hepatitis, diarrhea, and asymptomatic pancreatitis. Notably, no case of ICI-DM was reported. Envafolimab demonstrated a favorable safety profile with manageable irAEs (10). Similarly, a prospective, open-label, single-arm phase II clinical trial evaluated the safety and efficacy of envafolimab plus lenvatinib with transarterial chemoembolization (TACE) in 37 patients with unresectable hepatocellular carcinoma. Treatment-related adverse events (TRAEs) of grade 3 or higher occurred in 52.6% of patients. The most common grade 3 or worse TRAEs were increased aspartate aminotransferase (AST), increased alanine aminotransferase (ALT), and decreased platelet count. The increases in AST and ALT were mainly attributed to TACE. Only one patient experienced a serious AE (gastrointestinal bleeding), which required treatment discontinuation. No cases of hyperglycemia or diabetes were linked to the treatment (13). In a separate retrospective study of 64 patients with advanced malignant solid tumors treated with envafolimab reported adverse events classified as “hyperglycemia” in 15 patients(23.4%), though none fulfilled diagnostic criteria for diabetes. It should be noted that only 3 of these 64 received envafolimab monotherapy. Whether these hyperglycemic events were attributable to envafolimab remains uncertain (14). Liu et al. reported a case of combined SCLC who treated with carboplatin and etoposide concurrently with envafolimab. A comprehensive efficacy evaluation demonstrated a PR, and no treatment-related serious adverse events were observed such as ICI-DM or CIP (15). However, with expanding clinical use of envafolimab, some rare and serious adverse events are reported in case reports, such as type 1 diabetes mellitus presenting with DKA and localized skin necrosis (16, 17). Although envafolimab is generally well-tolerated, these findings highlight the potential for severe irAEs and underscore the need for close monitoring.

The National Comprehensive Cancer Network (NCCN), American Society of Clinical Oncology (ASCO), Chinese Society of Clinical Oncology (CSCO) and European Society for Medical Oncology (ESMO) clinical practice guidelines for the management of toxicities from immunotherapy have defined and graded ICI-related hyperglycemia but not ICI-DM (18–21). Nearly all reported cases of ICI-DM presented with rapidly elevated blood glucose due to the rapid β-cell destruction (22, 23). Therefore, some researchers suggest that low or undetectable fasting and postprandial C-peptide levels can serve as the diagnostic criteria of ICI-DM (24, 25). In our patient, despite negative diabetes-associated antibodies and normal C-peptide, the rapid clinical onset and appearance of DK following insulin cessation support the diagnosis of ICI-DM.

Current literature indicates that the pathogenesis of ICI-DM involves immune-mediated destruction of pancreatic β-cells, a process similar to that observed in spontaneous T1DM (25, 26). Specifically, Inhibiting PD-L1 can activate CD4+ and CD8+ T cells, leading to the release of pro-inflammatory cytokines such as interferon-γ and tumor necrosis factor-α (27). This cytokine surge promotes the recruitment of additional immune cells and induces β-cell apoptosis. Furthermore, the loss of PD-L1 signaling breaks immune tolerance against β-cells, amplifying the autoimmune response (28). However, the precise mechanisms remain to be fully elucidated in this context.

Several islet autoantibodies have been linked to ICI-DM, including GAD65, zinc transporter 8 autoantibodies(anti-ZnT8), IA-2A, IAA, and ICA. Among patients with positive islet antoantibodies, GAD65 is the most prevalent (39.4%, 37/94) and may be associated with the rapid onset of ICI-DM (25). However, some studies have reported cases without traditional autoimmune markers such as GAD65 or IA-2A, suggesting the possibility of an alternative mechanism induced by ICI therapy (29). This mechanism is different from classical T1DM. In our case, all autoimmune markers were negative, the absence of these markers does not exclude a diagnosis of ICI-DM, as some patients may present with a unique immunological profile following ICI treatment.

A retrospective cohort study involving 109 documented cases of ICI-DM showed that the majority of patients were male, with a mean age of 62 years. The onset of ICI-DM occurred as early as 1 week and as late as 26 months after initial ICI treatment, with a median onset time of 13 weeks. Envafolimab was notably absent from this analysis, likely due to the scarcity of reported cases associating it with diabetes (11). In our case, the time of ICI-DM onset was 8 months after envafolimab initiation, indicating a significantly delayed manifestation compared to the findings reported by Zhou et al. This is consistent with the later onset of ICI-DM observed in autoantibody-negative patients (11).

Glycemic control was achieved and envafolimab was continued. Subsequently, CIP occurred at 19 months after envafolimab initiation, leading to permanent discontinuation. Similarly, Liu et al. reported a case of extensive-stage SCLC in which DKA emerged 2 months after initiation of CS1003(an anti-PD-1 monoclonal antibody, Cstone Pharmaceuticals, China), followed by immune-related pneumonitis at 15 months. That patient maintained a PR for 2 years (12). In our case, metastasis to the left submandible developed at 15 months of therapy. Envafolimab was discontinued at 19 months. To date, apart from progression in the left submandibular region and adjacent lymph nodes, all other lesions remain in PR. Both our case and the report by Liu et al. describe SCLC patients treated with PD-1/PD-L1 inhibitors who developed ICI-DM and CIP, yet achieved favorable disease control. In a comprehensive review of ICI-DM prognosis, tumor response was assessed in 70 cases: 11(15.7%) showed complete response (CR), 39(55.7%) PR, 10(14.3%) stable disease (SD). Although these case series included different kinds of cancer, 71.4%(50/70) of patients with ICI-DM achieved an objective response-a rate significantly higher than that reported in other phase 3 immunotherapy clinical trials (9). These findings provide new evidence linking ICI-DM with improved clinical outcomes.

In our case, envafolimab was permanently discontinued due to CIP, whereas Liu et al. reported only temporary suspension of immunotherapy. This indicates that immunotherapy rechallenge may be feasible in subsequent lines of treatment and supports the safety of resuming immunotherapy after glycemic control in ICI-DM. Given the limited number of cases, further validation is required to this hypothesis whether ICI-DM can serve as a predictive biomarker for a favorable prognosis. Importantly, these findings underscore the critical role of insulin therapy in managing ICI-DM; discontinuation of insulin can rapidly lead to DK or DKA.

## Data Availability

The original contributions presented in the study are included in the article/supplementary material. Further inquiries can be directed to the corresponding author.
